# WZB117 Decorated Metformin-Carboxymethyl Chitosan Nanoparticles for Targeting Breast Cancer Metabolism

**DOI:** 10.3390/polym15040976

**Published:** 2023-02-16

**Authors:** Anindita De, Ashish Wadhwani, Sauraj, Parikshit Roychowdhury, Ji Hee Kang, Young Tag Ko, Gowthamarajan Kuppusamy

**Affiliations:** 1 College of Pharmacy, Gachon Institute of Pharmaceutical Science, Gachon University, Incheon 21936, Republic of Korea; 2 Faculty of Health Sciences, School of Pharmacy, JSS Academy of Higher Education and Research, Mauritius, Droopnath Ramphul St, Vacoas-Phoenix 73304, Mauritius; 3 Department of Pharmaceutics, JSS College of Pharmacy, JSS Academy of Higher Education and Research, Ooty 643001, Tamil Nadu, India

**Keywords:** breast cancer metabolism, metformin, WZB117, conjugation, in vitro cancer efficacy

## Abstract

The “Warburg effect” provides a novel method for treating cancer cell metabolism. Overexpression of glucose transporter 1 (GLUT1), activation of AMP-activated protein kinase (AMPK), and downregulation of mammalian target of rapamycin (mTOR) have been identified as biomarkers of abnormal cancer cell metabolism. Metformin (MET) is an effective therapy for breast cancer (BC), but its efficacy is largely reliant on the concentration of glucose at the tumor site. We propose a WZB117 (a GLUT1 inhibitor)-OCMC (O-carboxymethyl-chitosan)-MET combo strategy for simultaneous GLUT1 and mTOR targeting for alteration of BC metabolism. WZB117 conjugated polymeric nanoparticles were 225.67 ± 11.5 nm in size, with a PDI of 0.113 ± 0.16, and an encapsulation of 72.78 6.4%. OCMC pH-dependently and selectively releases MET at the tumor site. MET targets the mTOR pathway in cancer cells, and WZB117 targets BCL2 to alter GLUT1 at the cancer site. WZB117-OCMC-MET overcomes the limitations of MET monotherapy by targeting mTOR and BCL2 synergistically. WZB117-OCMC-MET activates AMPK and suppresses mTOR in a Western blot experiment, indicating growth-inhibitory and apoptotic characteristics. AO/EB and the cell cycle enhance cellular internalization as compared to MET alone. WZB117-OCMC-MET affects cancer cells’ metabolism and is a promising BC therapeutic strategy.

## 1. Introduction

Breast cancer (BC) is one of the leading causes of death among women, but it can also affect men [1]. BC has now been categorized as a genetic metabolic pathway disorder [2,3]. Otto Heinrich Warburg introduced the term “Warburg effect” to describe the metabolic shift of cancer cells from mitochondrial oxidative phosphorylation (OXPHOS) to aerobic glycolysis [4,5]. Cancer cells accelerate the effects of aerobic glycolysis to meet the enormous energy demand for rapid cell division and survival. This alternative metabolism of cancer cells opens the door to novel therapeutic options [6]. The key energy regulator during cancer cell division is adenosine monophosphate-activated protein kinase (AMPK), which may regulate energy balance at the cellular and whole-body levels [7]. AMPK is an important link in the metabolic and signaling networks that acts as a metabolic tumor suppressor by influencing energy levels, implementing metabolic checkpoints, inhibiting cell growth, and downregulating the mammalian target of rapamycin (mTOR) pathway. Inhibiting mTOR is responsible for the inhibition of cell growth, cell cycle progression, cell proliferation, and cancer arrest. So, AMPK and mTOR might be potential diagnostic and therapeutic biomarkers for BC.

Metformin (MET) (anti-diabetic drug), 5-aminoimidazole-4-carboxamide ribose (AMPK pharmacologic activator), RL71 (curcumin analog), dimethoxy-curcumin (structural analog of curcumin), Fluoxetine (selective serotonin reuptake inhibitor), and OSU-53 (thiazolidinedione compound) are a few therapeutics which show excellent therapeutic activity for the activation of AMPK and blocking of the mTOR pathway. MET exhibited the safest profile and was the most effective AMPK activator for anticancer therapy among all of these pharmaceuticals [8,9,10,11]. MET activates AMPK and downregulates mTOR in tumor cells, which reduces protein and lipid synthesis and restricts cancer cell growth and proliferation [12] MET is a powerful anticancer agent that inhibits cell proliferation and induces apoptosis. However, due to its low bioavailability and short half-life, MET monotherapy is limited, and its therapeutic effectiveness is dependent on glucose levels at the tumor site [13]. Under high glucose conditions, MET suppresses proliferation but does not induce apoptosis. As a result, reducing the glucose level at the tumor site is necessary for MET to carry out effective anti-proliferation and apoptosis functions to provide effective cancer management. Reducing the extracellular glucose level of cancer cells by combining MET with a glucose receptor inhibitor may therefore be beneficial in boosting MET’s therapeutic potential [14,15].

**Figure 1:**
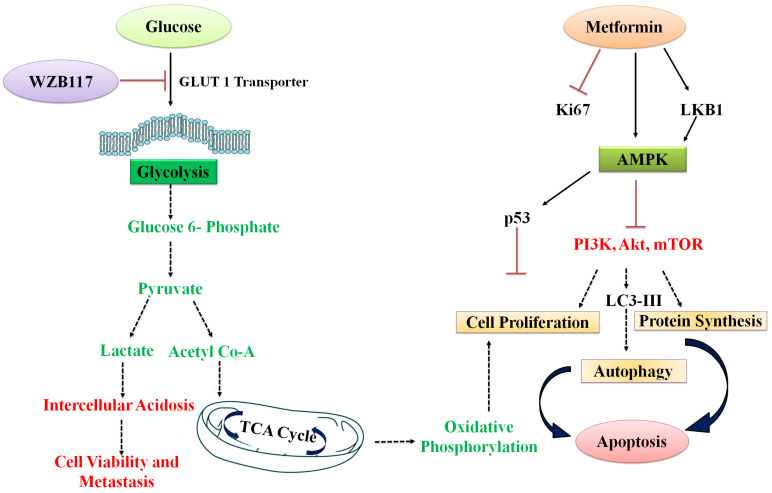
Combination of metformin and WZB117 for anti-cancer therapy: WZB117 inhibit the glucose transport to the cancer cells and create the energy stress by targeting extracellular GLUT1 pathway. Metformin inhibits the cell proliferation and protein synthesis by blocking mTOR, Akt, and PI3K pathway and induced apoptosis. The combination synergistically target GLUT1 and AMPK pathway to target the cancer cell apoptosis.

Glucose transporters (GLUT) are overexpressed in cancer cells to meet the increased glucose demand of onco-cells [16]. Most tissues in the body rely on GLUT1 for glucose absorption; however, it is overexpressed in cancer cells. As a result, these overexpressed GLUT receptors serve as oncogenes, and their overexpression has been associated with BC aggressiveness and a poor prognosis [17]. WZB117, a well-known GLUT1 inhibitor, inhibits glucose transport and cancer cell proliferation efficiently [18]. WZB117 effectively prevents glucose transfer from the extracellular to the intracellular compartment, resulting in energy stress and anti-proliferative activity. However, as monotherapy, it fails to trigger apoptosis. As a result, a WZB117-MET combination, in where WZB117 reduces glucose levels at the tumor site by inhibiting GLUT1 receptors and synergistically enhances MET therapeutic efficacy to induce apoptosis, might be a promising approach to target cancer cell metabolism. Figure 1 depicts WZB117-MET’s synergistic mechanism for altering cancer cell metabolism. 

A good carrier is necessary for high loading and to overcome the limitations of MET’s poor bioavailability and short half-life [13]. Chitosan and its derivatives are gaining popularity due to its cationic nature and endosomal escape, biodegradability, low cost, and therapeutic efficacy, particularly in BC [19,20,21]. O-carboxymethyl chitosan (OCMC) has received a lot of attention [22,23,24] for its ability to overcome chitosan’s water insolubility barrier while still retaining chitosan’s advantages for cancer cell targeting. Its reactive O-carboxymethyl substitution can react with the NH_3_^+^ group of MET, enhancing its entrapment and drug loading [25,26]. The formulation of nano-sized particles improves uptake, transport, and endosomal escape. The pH-specific MET release also has a favorable effect due to the pH sensitivity of OCMC, which releases MET exclusively in the tumor microenvironment. This is one of the first studies to target an alternative cancer metabolism for inhibiting cell growth and apoptosis pathway using the synergistic effects of MET, WZB117, and OCMC polymeric nanoparticles (Nps).

The method is applicable to the majority of malignancies (colon, lungs, ovarian, and glioblastoma) [15,27,28,29,30,31] where GLUTs are overexpressed and act as a biomarker. In this study, we specifically target BC metabolism to demonstrate the concept of the GLUT 1 inhibitors-MET combo’s synergistic effectiveness in targeting cancer cell metabolism.

## 2. Materials and Methods

### 2.1. Materials

Apex Laboratories, Chennai, India, kindly gifted Metformin. The 140–220 K OCMC was procured from Indian Sea Food Pvt. Ltd., Kerala India. Crosslinker calcium chloride (CaCl_2_) and cryoprotectant trehalose were obtained from Sigma-Aldrich, St. Louis, MO, USA. Acetonitrile and methanol were procured from S.D. Fine Chemicals, Mumbai, India. Dulbecco’s Modified Eagle Media (DMEM) was from Himedia, Mumbai, India. The MCF-7, MDA-MB-231, and MCF-10A cell lines were purchased from the National Centre for Cell Science (NCCS), Pune, India. All other chemicals used were of analytical grade.

### 2.2. Methods

#### 2.2.1. Formulation and Evaluation of OCMC-MET Nanoparticles 

The OCMC-MET polymeric Nps were prepared by ionic gelation technology. The polymer solution was pre-incubated with MET (20 mg) overnight at 1000–2000 rpm. The crosslinking agent, calcium chloride (2% CaCl_2_), was added into the OCMC solution dropwise until turbidity appeared. The resulting colloidal solution was homogenized by a high-speed homogenizer at 14,000 rpm for 6 min/3 cycles to formulate the OCMC-MET Nps. The formulation was centrifuged at 10,000 rpm, and the Nps were lyophilized using the cryo-protectant trehalose (5% *w/v*). Plackett–Burman Design (PBD) and Box–Behnken Design (BBD) were used to optimize the process parameters for OCMC-MET formulation [7,9]. The detailed optimization was published in our previous publication [9] and provided in Figure S1. 

MET-loaded polymeric Nps were evaluated for particle size (PS), poly-dispersibility index (PDI), and zeta potential (ZP) using dynamic light scattering (DLS) technology. All the DLS analyses were carried out at 25 °C in triplicate by the Malvern Zetasizer (Nano ZS, Malvern, Worcestershire, Instruments, UK) at an angle of 90° with a viscosity value of 0.898 cP and a refractive index of 1.332 for data point analysis [32]. The morphology of the OCMC-MET polymeric Nps was studied by scanning electron microscopy (SEM) (JEOL JSM 7610F, Tokyo, Japan) operated at 15 keV with different resolutions [25,26,33]. The ultra-fast liquid chromatography (UFLC) (Shimadzu LC2010A HT, Tokyo, Japan) method was used to estimate the entrapment efficiency (EE) of MET. The swelling index [22,34] was measured by soaking 20 mg of formulation in 50 mL of PBS at 37 °C with two different pH values of 5.5 (cancer microenvironment) and 7.4. (physiological) to understand the effect of pH on Nps. The OCMC-MET formulation was incubated for 1 h and 5 h to achieve swelling equilibrium. The degree of swelling was calculated by the following equation:

(1) $$\%\;Swelling\;Index=\frac{W_{s}-W_{d}}{W_{d}}\times 100$$

where *W_s_* was the weight of the swelled sample and *W_d_* is the wet sample weight. 

In vitro MET release was evaluated using a dialysis membrane analysis (weight cut off 12,000–14,000, HiMedia, Mumbai, India). A total of 10 mg of the OCMC-MET formulation was submerged in 100 mL of simulated cancer fluid (pH 5.5) or normal body fluid (pH 7.4) at 37 °C and 100 rpm. Throughout the investigation, the sink conditions were maintained.

#### 2.2.2. Synthesis and Characterization of WZB117-OCMC-MET 

WZB117 was esterified on the surface of OCMC-MET polymeric Nps using dicyclohexylcarbodiimide (DCC) as a catalyst. WZB117 and DCC were dissolved in 20 mL of dichloromethane and stirred for 8 h at room temperature. After that, the solution was dropwise added to 30 mg of OCMC-MET, which was dissolved in 100 mL of sodium bicarbonate solution (pH = 8.3). The blended mixture of OCMC-MET and WZB117 was fluxed for 14 h [35]. The final product was precipitated by an ice bath [36]. 

WZB117-OCMC-MET structural characterization was determined using FTIR and compared to the MET, OCMC, WZB117, and OCMC-MET spectra. For analysis, the material was formed into a pellet using KBr (1:1) at room temperature [37]. The mass spectra of MET, OCMC, WZB117, and WZB117-OCMC-MET were recorded in +VE ion electron mode (Shimadzu 8030 System, Tokyo, Japan). The mobile phase was water: methanol (20:80 *v*/*v*) solution, with a flow rate of 0.5 mL/min and an injection volume of 10 µL. The mass range of the compounds was recorded in scan mode from 10 to 800 *m*/*z*. The sample solutions were injected into liquid chromatography mass spectrometry/mass spectroscopy (LC-MS/MS) and the corresponding molecular ion peak was scanned. The appearance of any new fragment in the mass spectrum was recorded to confirm the conjugation in comparison to OCMC-MET.

### 2.3. In Vitro Cytotoxicity Study to Evaluate the Efficacy of Conjugate Nanoparticles 

The carcinoma cell line MCF-7, the highly aggressive TNBC cell line MDA-MB-231, and the non-carcinogenic cell line MCF-10A were used to investigate the therapeutic efficacy of WZB117-OCMC-MET. The EZ-Cytox Cytotoxicity Test Kit was used to evaluate the formulation’s cytotoxicity utilizing the 3-(4,5-dimethylthiazol-2-yl)-2,5-diphenyltetrazolium bromide (MTT) assay (DoGen Bio, Seoul, South Korea). Cancer cells were plated in a 96-well plate at a density of 2 × 10^5^ cells per well in 100 µL Dulbecco’s Modified Eagle Media (DMEM) and cultured for 24 h [38,39]. Following the incubation time, cells were washed with 1 mL of PBS and kept for 48 h at 37 °C under 5% CO_2_ conditions with different concentrations (1.0–100 µg/mL) of MET, OCMC-MET, WZB117-OCMC-MET, and WZB117. As in the control, DMSO at a 0.5% (*v*/*v*) concentration was used. The media was removed after 48 h, and the cells were rinsed in 1 mL of PBS. Following that, each well received 20 µL of MTT solution (stock: 5.0 mg/mL in PBS) from the EZ-Cytox Cytotoxicity Assay Kit. The cells were cultured for 4 h to allow mitochondrial dehydrogenases to activate. Finally, the absorbance of the formazan at 450 nm was estimated using a microplate reader (VICTORTM X3, PerkinElmer). The experiment was carried out three times. Nonlinear regression analysis and a dose-response curve were generated using GraphPad Prism 7.0 (GraphPad Software, Inc., San Diego, CA, USA) software to identify the concentration of each formulation that inhibited growth by 50% and the 95% confidence interval.

The Selectivity Index (SI) was calculated by dividing the cytotoxicity (IC_50_) in cancer cells (MCF-7 and MDA-MB-231) by the cytotoxicity (IC_50_) in normal cells (MCF-10A). SI values greater than 3 were found to be more cytotoxic to MCF-7 and MDA-MB-231 cells.

### 2.4. Therapeutic Efficacy of WZB117-MET Combination by Compusyn^®^ Software

The combination of therapies must conform to their synergistic efficacy through the combination index (CI) hypothesis as defined by Compusyn^®^ software ver. 1.0 (ComboSyn Inc., Paramus, NJ, USA). According to the theory, CI < 1, =1, and >1 designate synergistic, additive, and antagonistic effects, respectively [40]. Dose-reduction index (DRI) of =1, >1, and <1 indicate no dose reduction, favorable dose reduction, and unfavorable dose reduction. The combination dose was calculated using the Compusyn^®^ software using the formula:

(2) $$Combination\;index\;\left(CI\right)=\frac{{\left(A\right)}_{1}}{{\left(A_{x}\right)}_{1}}+\frac{{\left(A\right)}_{2}}{{\left(A_{x}\right)}_{2}}$$

(*A_x_*)_1_ and (*A_x_*)_2_ were concentrations of each drug alone that produced the therapeutic effect, whereas (*A*)_1_ and (*A*)_2_ were concentrations of drugs together that had the same effect.

(3) $$Dose\;Reduction\;Index\;\left(DRI\right)=\frac{{\left(A_{m}\right)}_{n}{\left[\frac{f_{a}}{1-f_{a}}\right]}^{1/m_{n}}}{{\left(D\right)}_{n}}$$

(*A_m_*)*_n_* is the median effect dose of the *n*th no of the sample, *A* is the dose, *f_a_* is the fraction affected individual half-maximal inhibitory concentration (IC_50_) values for different concentrations of MET and WZB117 which were determined in the cancer cell line cytotoxicity investigation and were used to compute the CI and *DRI* [41].

### 2.5. Colony Formation Assay to Evaluate the Long Term Efficacy of Conjugate Nanoparticles 

The clonogenic assay was used to investigate the long-term influence of cytotoxic compounds for 7 days. MDA-MB-231 cells were seeded at a density of 2000 cells/well in 6-well plates in triplicate. After washing the cells with 1 mL PBS, MET, OCMC-MET, WZB117-OCMC-MET, and WZB117 were added and cultured for 7 days at 37 °C with 95% air and 5% CO_2_. The supernatant was removed and rinsed with 2 mL PBS after 7 days of incubation. The cells were fixed in a 3:1 mixture of methanol and formaldehyde, and the colonies were stained with 0.5% crystal violet for 15 min. The excess pigment was removed with distilled water. A microscope was used to count colonies with more than 50 cells. Cell survival after long-term therapy with the MET, OCMC-MET, WZB117-OCMC-MET, and WZB117 was calculated using the number of colonies [25].

### 2.6. Cell Morphology Alteration Assay by AO/EB Staining to Evaluate the Efficacy of Conjugate Nanoparticles

MET, OCMC-MET, WZB117, and WZB117-OCMC-MET were tested for their effect on apoptosis using two distinct stains, acridine orange (AO) and ethidium bromide (EB) [42]. Cells were grown in T25 flasks with a final concentration of 2 × 10^4^ cells/mL in complete DMEM media. The cells were treated with an 8.3 µg/mL concentration of MET and an equivalent concentration of MET in the OCMC-MET and WZB117-OCMC-MET formulations for 48 h. The cells were trypsinized (600 µL) and centrifuged at 1500 rpm for 3 min to collect the cells. After washing with 1 mL PBS, the suspension was re-dispersed in 1 mL of DMEM solution. The cells were then stained for 15 min at 37 °C with an AO/EB combination (10 μL) containing 5 μg/mL AO and 5 μg/mL EB (AO/EB, Sigma, St. Louis, MO, USA). To prevent additional dye penetration into cells, ice-cold PBS was used for washing. The cell pellets were obtained by centrifugation at 1000 rpm for 4 min. Cell pellets were re-dispersed in PBS and examined for apoptotic cells using a phase contrast microscope (Hund, Germany). At least 300 cells in each of the four fields were observed for viability, early and late apoptosis, and necrosis. The experiment was performed in triplicate. Cells without treatment served as controls.

### 2.7. DNA Fragmentation Analysis to Evaluate the Efficacy of Conjugate Nanoparticles 

DNA fragmentation was investigated as a qualitative biomarker of cell death. MDA-MB-231 cells treated with OCMC-MET, WZB117, and WZB117-OCMC-MET were compared to untreated MDA-MB-231 cells using the DNA ladder as a marker. Following the treatment, 5 × 10^6^ cells/mL were transferred to a lysis buffer at 65 °C [43]. The DNA was extracted using phenol/chloroform/isoamyl-alcohol (25:24:1 *v*/*v*). The DNA was re-suspended in Tris-EDTA buffer, pH 8.0, at 37 °C for 1 h. The extracted DNA was mixed with 1× loading dye and run in triplicate on a 1% agarose gel at 100 V for 15 min. The fragmented DNA was imaged using a UV transilluminator (Bio-rad, Hercules, CA, USA).

### 2.8. Apoptosis Assay to Evaluate the Efficacy of Conjugate Nanoparticles by Flow Cytometry 

MDA-MB-231 cells (1 × 10^5^ cells/well) were plated in 6-well plates for 24 h in DMEM media. The cells were treated with an 8.3 µg/mL concentration of MET and an equivalent concentration of MET in the OCMC-MET and WZB117-OCMC-MET formulations. Treated cells were trypsinized, collected, and then centrifuged at 15,000 rpm for 3 min. Cells were washed twice with 1 mL of PBS. Cells were stained with 5 µL of annexin V-FITC and 10 µL of Propidium iodide (PI) and incubated for 45 min in the dark. The FAC Scan flow cytometer can distinguish between living, necrotic, early apoptotic, and late apoptotic cells (Beckton Dickinson Accuri C6). The % of annexin V-positive cells was used to calculate the degree of apoptosis.

### 2.9. mTOR and BCL2 Downregulation Assay by Western Blotting to Evaluate the Efficacy of Conjugate Nanoparticles

BC is a metabolic condition that is caused by changes in the expression of the proteins AMPK and mTOR, as well as the glycolysis protein BCL2 [43]. MAD-MB-231 (1 × 10^5^ cells/well) cells were grown in 6-well plates and subsequently treated for 24 h with OCMC-MET, WZB117, and WZB117-OCMC-MET. Following treatment, a radioimmunoprecipitation assay buffer was used to lyse the cells. The cells were centrifuged (12,000× *g*, 30 min) at 4 °C, and the supernatant were collected. The recovered material was used to electrophorese the proteins. Bradford’s technique was used to determine the total protein concentration. Proteins (30 g/lane) were electrophoresed in 10% SDS-PAGE before being transferred to PVDF membranes. These membranes were then treated overnight at 40 °C with primary antibodies against AMPK (1:200), pAMPK (1:1000), mTOR (1:1000), BCL2 (1:200), and GAPDH (1:200). An amount of 1 mL of TBST buffer was used for washing. PVDF membranes were incubated for 1 h with the secondary antibody, horseradish peroxidase-conjugated goat anti-rabbit IgG. The downregulation of mTOR and BCL2 and the upregulation of AMPK were detected using 0.01% 3,3′-diaminobenzidine to predict the efficacy of WZB117-OCMC-MET compared to OCMC-MET and WZB117.

### 2.10. Cellular Uptake Assay by Confocal Microscopy to Evaluate the Efficacy of Conjugate Nanoparticles

Since MET is a nonfluorescent drug, formulations of OCMC-MET and WZB117-OCMC-MET with cysteine 5.5 (Cy5.5) coating were developed to examine cellular uptake. A total of 50 mg of the formulations were dissolved in 10 mL of distilled water (DI), and then 0.5 mg of Cy5.5 in DMSO was added dropwise. After complete solubilization, EDC and NHS were added and incubated for 5 h in the dark at room temperature with continuous stirring (500 rpm) at pH 5.5. Dialysis (MW cut-off = 2.5 kDa) was performed for 48 h to get the Cy5.5-conjugated OCMC-MET. The final product was lyophilized and stored at 8 °C until use. The Cy5.5-conjugated OCMC-MET was used to produce the WZB117-OCMC-MET [44]. 

MDA-MB-231 cancer cells were cultured in a Petri dish at 1 × 10^4^ for 48 h. Cy5.5-labeled WZB117-OCMC-MET was added to the Petri plate and incubated for 2 h at 37 °C. Cells were washed with 1 mL of PBS before being fixed with 0.4% *w/v* formaldehyde at 4 °C. The cells were incubated in DMEM with 4′,6-diamidino-2-phenylindole (DAPI) in a cold and dark place until imaging [45]. A CLSM (Carl Zeiss CLSM-710, Oberkochen, Germany) was used to photograph the cells. The fluorescence of the Cy5.5-labeled Nps was measured using a confocal microscope at 650 nm for excitation and 675 nm for emission.

### 2.11. Cell Cycle Assay to Evaluate the Efficacy of Conjugate Nanoparticles

The flow cytometer was used for the cell cycle analysis. MDA-MB-231 cells were planted in 6-well plates (1 × 10^5^ cells/well) and incubated for 24 h. After harvesting, the cells were washed with 1 mL PBS and treated with an 8.3 µg/mL concentration of MET and an equivalent concentration of MET in the OCMC-MET and WZB117-OCMC-MET formulations for 24 h. The trypsinized cells were then rinsed with PBS and fixed with 1 mL of ice-cold 70% ethanol. Finally, cells were treated for 45 min with 25 µg/mL PI and 200 mg/mL RNase A. The control (paclitaxel) was incubated separately and set aside as well. DNA content was measured using a forward scatter (FAC). Scan flow cytometer (Becton Dickinson Accuri C6, San Jose, CA, USA) was used to determine the samples after 48 h.

## 3. Results and Discussion 

### 3.1. Formulation and Evaluation of OCMC-MET Nanoparticles 

**Figure 2:**
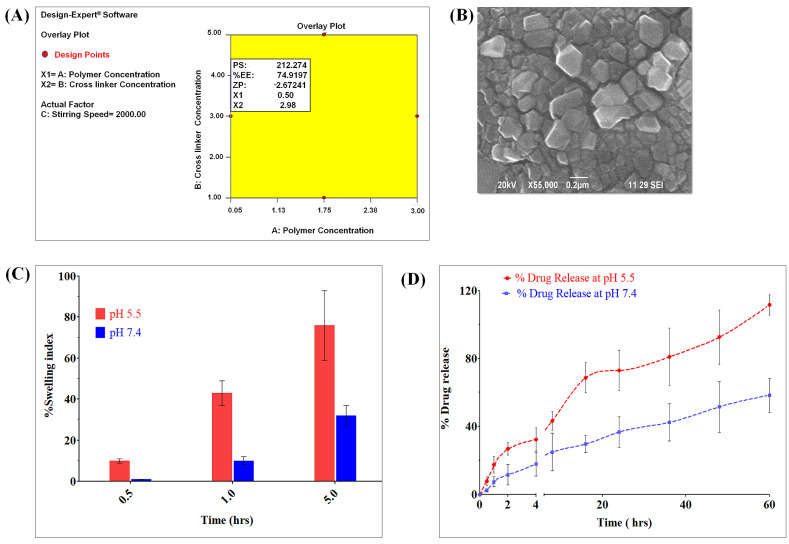
(**A**) Overlay plot obtained for the OCMC-MET polymeric nano-formulation optimized by the DoE approach. (**B**) SEM morphological study of OCMC-MET at 0.2 µm scale showed that due to high charges the aggregated spherical of OCMC-MET Nps. (**C**) Swelling index of the polymeric Nps in different pH after 0.5 h, 1 h, and 5 h. (**D**) In vitro MET release from OCMC polymeric Nps in two different pH conditions (*n* = 3).

OCMC-MET polymeric Nps were prepared by ionic gelation technology. The methylation of chitosan increases the OCMC polymer’s solubility [46]. The OCMC’s anionic carboxymethyl group interacted with the MET’s cationic amine group to increase drug accumulation and therapeutic efficacy [22,38,46]. CaCl_2_ improves mechanical strength through an electrostatic bond. These interactions originate from the adiabatic proton transfer procedure [47,48]. At different pH levels, opposite charges govern the degree of ionization and the intensity of interactions. The ionization also indicates that OCMC Nps cross-linked with Ca^+^ has pH-sensitive properties [48]. The OCMC carboxylic groups (pKa = 3.40) are largely deprotonated at pH 5.5. If the interaction of Ca^2+^ ions with COO groups is the fundamental mechanism involved in the formation of Nps, the amount of Ca^2+^ ions required should be relatively constant. Because the concentration of Ca^2+^ ions required for polymeric Nps formation is low at pH 5.5, the interaction between the −COO and NH_3_+ groups is more critical for NPs formation at higher pH than the interaction between the Ca+ and −COO. The amino groups’ pKa value was around 7.15, indicating that half of the amino groups are protonated at this pH, confirming the hypothesis of cancer cell-specific drug release and a reduction in swelling at a lower pH [46,49,50]. The previous research showed [46,50,51] that lower pH conditions where protonated amine groups of OCMC interact instantaneously with anionic groups of OCMC through electrostatic attraction to form crosslink Nps. Our previous study discussed the DoE (Figure S1 and Figure 2A) method for optimizing the OCMC-MET formulation in depth [9].

The optimum OCMC-MET polymeric Nps had a PS of 225.67 ± 11.5 nm, an %EE of 72.78 ± 6.4%, and a ZP of −2.22 mV with PDI of 0.113 ± 0.16 (Figure S2). The SEM image (Figure 2B) indicated spherical particle formation, but they were prone to aggregate due to high specific surface energy [37,46,50]. The pH-reactive property of OCMC might be linked to the protonation of the functional amino group, which results in increased electron density between cross-linked OCMC chains. As a result, it was considered that formulated OCMC-MET polymeric Nps had effective drug release in a cancer microenvironment. 

With increasing pH levels, the swelling ratio OCMC-MET increased. This was mostly attributable to the presence of a carboxylic group in the polymeric network as well as the amine group of the MET. As the pH of the solution increased, the amine group of the MET became ionized, resulting in static repulsion among the ionized groups. As a result, the swelling ratio of polyelectrolyte complexes achieves a relatively high value. Furthermore, in aqueous or alkaline conditions, these carboxylate anions (−COO) in the polymeric network have a higher solvation propensity than non-ionic groups. As a result, higher swelling has been reported at pH 7.4 as compared to pH 5.5. The % of swelling of OCMC-MET after 0.5 h was approximately 10.33 ± 1.1% at pH 5.5 and 1.5 ± 0.4% at pH 7.4. After 5 h the swelling of the formulation was around 76.66 ± 16.8% for pH 5.5 and only 32.23 ± 5.6% for pH 7.4 (Figure 2C, Table S1). The swelling is directly proportional to the drug release. The swelling ratio of the OCMC-MET is higher at pH 7.4, indicating a poor release of MET in the body fluid compared to the acidic cancer microenvironment.

The MET release from the OCMC-MET formulation was pH-dependent due to the pH-sensitive OCMC polymer-MET complex. The protonation of the OCMC and MET in different pH conditions due to their differing pKa values was helpful for the endosomal escape and release of the drug MET in the cancer microenvironment at pH 5.5. MET was released from the polymeric Nps by erosion, swelling, and diffusion, resulting in increased drug bioavailability and a prolonged therapeutic impact (Figure 2D). Following the initial erosion, the OCMC-MET formulation released MET via swelling-based and, at last, diffusion-based mechanisms. All things considered, 100 ± 8.9% MET release was observed at pH 5.5 at 60th hours, whereas only 58.4 ± 10.5% release was found at pH 7.4, which indicated the pH-specific drug release of the OCMC-MET formulation due to the OCMC polymer-MET complex.

#### Synthesis and Characterization of WZB117-OCMC-MET

**Figure 3:**
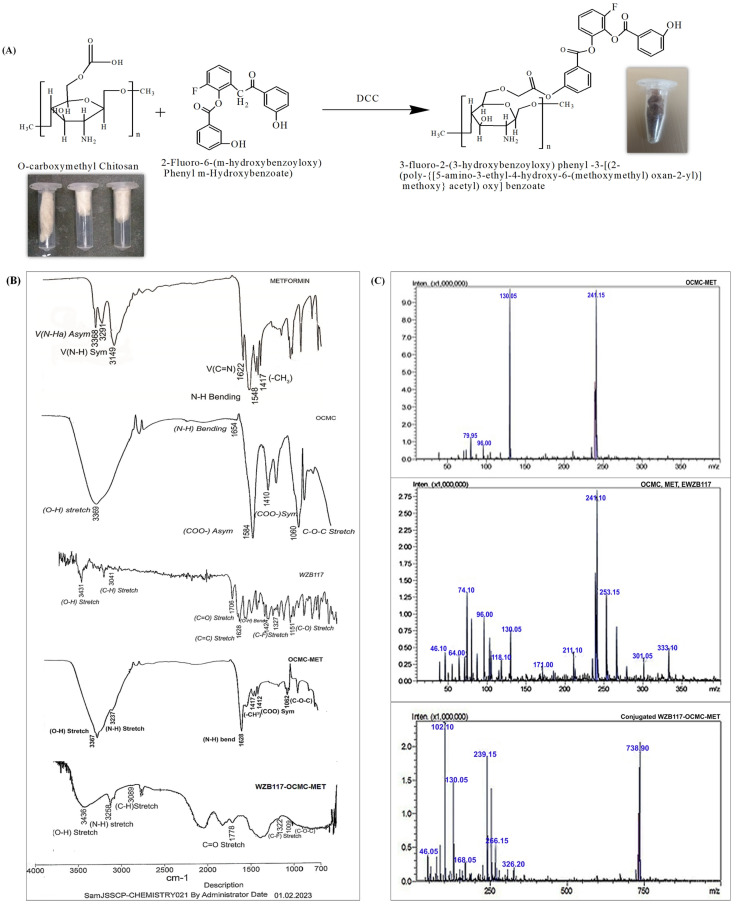
(**A**) Synthesis scheme of WZB117-OCMC-MET by a mono-esterification process to conjugate WZB117 on the surface of OCMC-MET. (**B**) FTIR spectrum Metformin, OCMC, WZB117, OCMC-MET, and WZB117-OCMC-MET to confirm the conjugation. (**C**) Mass spectroscopy of MET, OCMC, WZB117, and the WZB117-OCMC-MET conjugated formulation to confirm the conjugation process.

Figure 3A demonstrates the mono-esterification of the WZB117 (2-Fluoro-6-(m-hydroxybenzoyloxy) Phenyl m-Hydroxybenzoate) grafted-OCMC copolymer using DCC as a catalyst. The conjugated WZB117 is a selective biomarker for overexpressed transmembrane GLUT1 protein, which selectively binds to the extracellular GLUT1 receptors and reduces the glucose concentration by preventing facilitated diffusion of glucose inside the cancer microenvironment. The conjugation process of WZB117 with the OCMC-MET was verified by FTIR and mass spectroscopy. After encapsulating MET, the final product, 3-fluoro-2-(3-hydroxybenzoyloxy)-phenyl-3-[(2-(poly [29]acetyl)-oxy]-benzoate, is a novel complex formulated after the esterification process.

Figure 3B shows the FTIR spectra of MET, OCMC, WZB117, OCMC-MET, and WZB117-OCMC-MET. The spectra clearly showed the characteristic peaks of MET as 3368 cm^−1^ for N-H, 1622 cm^−1^ for C=N, 1548 cm^−1^ N-H bending, and 1417 cm^−1^ band peak for –CH_3_. The OCMC FTIR spectra show O-H at 3369 cm^−1^, N-H at 1654 cm^−1^, COO at 1410 cm^−1^, and the C-O-C band peak at 1060 cm^−1^. The GLUT1 inhibitor exhibited its typical peak at 1424 cm^−1^ for the C-F, 1706 cm^−1^ for the C=O bond, and 1151 cm^−1^ for the C-O bond. The band at 1417 cm^−1^ for OCMC-MET could be assigned to the stretch vibration of COO-, 3547 cm^−1^ for the aromatic alcohol stretching of the OH group along with the MET peaks, as the absorption peak at 3367 cm^−1^ was the secondary amine stretching vibration, and 1634 cm^−1^ was assigned to the N-H bending vibration without much change. In the WZB117-OCMC-MET polymeric formulation appearance of 1778 cm^−1^ for the C=O ester band indicate the esterification method. All the other characteristic peaks for the OCMC, MET, and WZB117 are also clearly present in the WZB117-OCMC-MET FTIR spectrum (Figure 3B). All of the results above indicate that WZB117 grafted OCMC-MET has been synthesized successfully. The mass spectra *m*/*z* of MET, OCMC, and WZB117 were 130, 241, and 333, respectively. The mass spectra of the WZB117-OCMC-MET conjugate revealed a molecular ion with *m*/*z* = 738. The conjugate had the same mass as the parent molecules. The appearance of a molecular ion signal at 738.90 confirmed the conjugate’s process (Figure 3C).

### 3.2. In Vitro Cytotoxicity Study to Evaluate the Efficacy of Conjugate Nanoparticles

**Figure 4:**
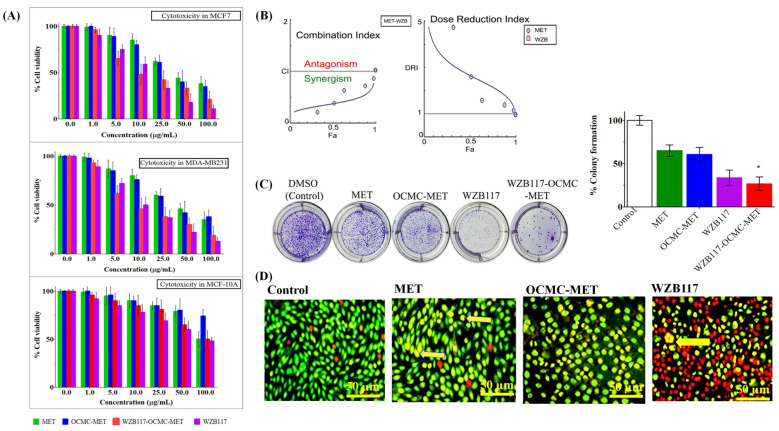
(**A**) Cytotoxicity studies in MCF7, MDA-MB-231, and MCF10A cell lines in vitro. (**B**) Graphical representation of CompuSyn^®^ software data for MET and WZB117 CI and DRI plots. (**C**) MDA-MB-231 cell line colony formation assay 1. Untreated cells (control); 2. cells treated with MET; 3. cells treated with OCMC-MET; 4. cells treated with WZB117; and 5. cells treated with WZB117-OCMC-MET. The graphical representation indicated the % reduction of the colony formation compared to the control group (*p* < 0.01) after the treatment. (**D**) Morphological changes of the MDA-MB-231cancer cells under treatment by MET, OCMC-MET, WZB117, and WZB117-OCMC-MET by AO/EB co-staining. Cells were observed under 10× and 20× magnifications by fluorescence microscopy (50 µm scale bar).

Different concentrations of MET, WZB117, OCMC-MET, and WZB117-OCMC-MET were studied for cancer cell growth (Figure 4A) for 48 h. A high concentration of MET and OCMC-MET were needed to inhibit cancer cell proliferation in the absence of WZB117 (GLUT1 inhibitor) (*p*-value < 0.001) indicating that the glucose concentration of the cancer cells microenvironment plays a significant role in cancer cell proliferation. In combination with WZB117-OCMC-MET, however, a low dosage of the compounds was enough to significantly restrict cancer cell growth, proving that the decoration of OCMC-MET with WZB117 synergistically enhanced the therapeutic efficacy of MET at a lower dose.

The selectivity index (SI) in Table 1 shows that the cytotoxic effects of MET, OCMC-MET, WZB117-OCMC-MET, and WZB117 were selected for MDA-MB-231 cells. The conjugated polymeric formulation had a higher SI score of 11.2 for the MDA-MB-231 cell line compared to 9.1 for MCF7 cancer cell lines. 

WZB117 conjugated with OCMC-MET enhanced cytotoxicity in cancer cells even in a high glucose cancer microenvironment (25 mM) compared to normal cell glucose conditions (5.5 mM) (data not shown in the article). WZB117 effectively reduced the glucose concentration at the cancer site by transcellularly binding with the GLUT1 inhibitor and reducing the glucose concentration inside the cancer cells. The OCMC’s positively charge assist to release the MET exclusively inside the cancer cells. A comparatively high concentration of MET was able to accumulate inter-cellularly and inhibit the mTOR pathway for cancer cell death. To prove the concept, we have performed further cellular internalization and apoptosis studies. WZB117-OCMC-MET was found to be non-toxic to the MCF10A cells. The tentative reason for the non-toxicity of the conjugated formulation may be the pH-specific cleavage of the ester bonds of the conjugated WZB117-OCMC-MET only in the cancer microenvironment due to highly acidic conditions. Subsequently, the IC_50_ value of MET, OCMC-MET, WZB117-OCMC-MET, and WZB117 for further study is shown in Table 1 for the highly selective MDA-MB-231 cell line. 

### 3.3. Therapeutic Efficacy of WZB117-MET Combination by Compusyn^®^ Software

The Compusyn^®^ program, version 1.0 (ComboSyn, Inc. Paramus, NJ, USA) was used to estimate the type of interaction between MET and WZB117 in combination. The median-effect strategy was applied to study the precise nature of the interaction between MET and WZB117. The current study found that combining MET and WZB117 had a synergistic growth inhibitory effect. As demonstrated in the Fa-CI plot (Figure 4B), the CI value ranges from 0.849 to 0.705 for fa = 0.6 to 0.9 [52], which was <1, clearly indicating the synergism of the combination therapy. The plot of DRI and fa (Figure 4B) also indicated a beneficial dosage reduction. The DRI for the combination was >1, indicating that the combination treatment led to a favorable dosage reduction. Despite the fact that MET and WZB117 have fundamentally different anticancer mechanisms, combined therapy has been shown to be synergistic in terms of anti-proliferative and cell death. The CI and DRI values generated from the Compusyn^®^ program demonstrated the required characteristics of high effectiveness with minimal toxicity, indicating an ideal combination. The in vitro cytotoxicity investigation was also very well correlated with the Compusyn^®^ software data. 

### 3.4. Colony Formation Assay to Evaluate the Long Term Efficacy of Conjugate Nanoparticles

MDA-MB-231 cells treated with MET, OCMC-MET, WZB117, and WZB117-OCMC-MET demonstrate a decrease in colony formation compared to the control group (consider 100% colony formation) (Figure 4C), providing evidence of long-term therapeutic effects of the conjugated formulation. A set of colony formation results from MDA-MB-231 cells is shown in Figure 4C, and quantitative data is in graphical form. WZB117-OCMC-MET had fewer colonies than OCMC-MET (*p* < 0.01), showing that conjugated formulations might limit proliferation and colony formation better than MET, OCMC-MET, and WZB117 over a prolonged time. The WZB117′s energy stress may be the principal cause of the WZB117-OCMC-MET long-term effects. Reduced glucose availability for cancer cells as a result of WZB117 conjugation increases MET’s thermogenic activity, resulting in energy depletion, which inhibits growth and contributes to cancer cell apoptosis. The efficacy of the MET and OCMC-MET was found to be very similar, indicating that the formulation with OCMC does not alter the therapeutic efficacy of the MET.

### 3.5. Cell Morphology Alteration Assay by AO/EB Staining to Evaluate the Efficacy of Conjugate Nanoparticles

During the AO/EB staining study, four distinct morphological characteristics of apoptosis were observed (Figure 4D). The AO, consumed by both live and dead cells, colored the DNA green. The EB stain was absorbed by dead cells and stained DNA a brilliant orange. Normal nuclei chromatin was labeled bright green for control cells, whereas viable apoptotic nuclei were labeled bright yellowish-greenish and orange to indicate the early and late stages of apoptosis with condensed or fragmented chromatin, respectively. A very small number of non-viable cells with normal nuclei stained in green were found in the MET and OCMC-MET formulations. MET and OCMC-MET treatments clearly indicated early-stage programmed cell death with granular yellow-green AO staining and a change in cell morphology. The staining was asymmetric, indicating that the cancer cells had survived apoptosis after being treated with MET and OCMC-MET. Brilliant orange staining of dead DNA treated with WZB117-OCMC-MET revealed late apoptotic cancer cells. WZB117-OCMC-MET treatment significantly increased early-stage apoptosis with asymmetrically localized orange nuclear EB staining. Necrosis cells were found in the periphery after WZB117-OCMC-MET treatment. WZB117 also showed late-stage apoptosis, but the number of necrotic cells was predominant, indicating the toxicity of WZB117, which might be a severe side effect of the WZB117 monotherapy.

### 3.6. DNA Fragmentation Analysis to Evaluate the Efficacy of Conjugate Nanoparticles

**Figure 5:**
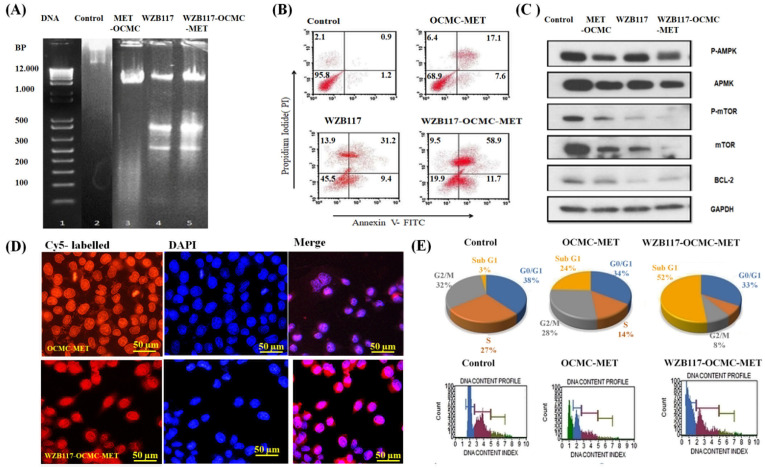
(**A**) DNA fragmentation of the MDA-MB-231 cell line using the MET-OCMC, WZB117, and WZB117-OCMC-MET. (**B**) Quantitative measurement of apoptosis by AnnexinV-FITC versus PI in MDA-MB-231 cells treated with OCMC-MET, WZB117, and WZB117-OCMC-MET. The data clearly indicate high late-phase apoptosis for the WZB117-OCMC-MET compared to OCMC-MET. (**C**) mTOR and BCL2 mediated apoptosis induced by WZB117-OCMC-MET compared with OCMC-MET. The OCMC-MET only downregulates the mTOR but does not alter the BCL2, whereas WZB117-OCMC-MET synergistically targets mTOR and BCL2. (**D**) Cellular internalization of WZB117-OCMC-MET and OCMC-MET in the MDA-MB-231 cancer cell line indicates that the complex was more effective in inter-cellular penetration due to the presence of WZB117 conjugation (50 µm scale bar). (**E**) Percentage population of the cancer cells and flow cytometric analysis at a different phase of the cell cycle.

After 24 h, the DNA was extracted, and the analysis was performed using agarose gel electrophoresis (Figure 5A). The results clearly showed that MDA-MB-231 cell lines treated with WZB117 and WZB117-OCMC-MET exhibited more DNA fragmentation than the marker. Faint fragmentation has also been observed in OCMC-MET therapy. As in previous cell line studies, MET and OCMC-MET showed very similar results, so we have taken OCMC-MET for further studies. MET may cause DNA fragmentation by directly blocking the mTOR pathway and depleting energy, leading to low ATP levels in cancer cells and DNA damage. WZB117 and WZB117-OCMC-MET had more band fragmentation. The reason may be that WZB117-OCMC-MET had synergetic activity due to the WZB117 conjugation with OCMC-MET. MET and WZB117 induce apoptosis synergistically by creating energy stress and mTOR inhibition more effectively and at a lower dose compared to MET monotherapy. WZB117-OCMC-MET displayed enhanced therapeutic efficacy, which could be attributable to the conjugation of the GLUT1 inhibitor, which boosts the therapeutic efficiency of the MET synergistically, as previously reported in CompuSyn^®^ data.

### 3.7. Apoptosis Assay to Evaluate the Efficacy of Conjugate Nanoparticles by Flow Cytometry

The Annexin V-FITC/PI double labeling was applied to quantify the polymeric Nps apoptotic effect on the MDA-MB-231 cancer cell line (Figure 5B). The fluorescent dye, PI, entered necrotic cells but not living cells. Detecting apoptosis phase cells using different staining patterns: viable (Annexin V− and PI−, lower left square), early apoptotic (Annexin V+ and PI−, lower right square), late apoptotic (Annexin V+ and PI+, upper right square), and necrotic (Annexin V− and PI+, upper left square) [53]. Figure 5B showed that the use of WZB117 and WZB117-OCMC-MET enhanced the number of late apoptotic cells compared to OCMC-MET. The control group had 1.2% early and 0.9% late apoptotic cells, whereas the WZB117-OCMC-MET group had 11.7% and 58.9%. WZB117 had 9.4% and 31.2%, and OCMC-MET had 7.6% and 17.1% of early and late apoptosis, respectively. Inhibition of mTOR by OCMC-MET causes late-stage apoptosis. In the case of the WZB117-OCMC-MET, it suppresses both BCL2 and mTOR due to the presence of WZB117 and MET. The combination of both MET and WZB117 exhibits synergistically increased late-stage apoptosis owing to glycolysis and oxidative energy stress suppression when compared to OCMC-MET alone. WZB117 was also found to be effective in producing apoptosis.

### 3.8. mTOR and BCL2 Downregulation Assay by Western Blotting to Evaluate the Efficacy of Conjugate Nanoparticles

For anticancer apoptosis activity, MET activates AMPK and downregulates mTOR, and WZB117 inhibits GLUT1, which inhibits BCL2 for apoptosis activity. We used p-AMPK, p-mTOR, and BCL2 to demonstrate the apoptosis pathway of WZB117-OCMC-MET. WZB117-OCMC-MET was able to induce AMPK phosphorylation levels in MDA-MB-231 cells, resulting in a decrease in mTOR and it downregulates BCL2 simultaneously, as shown in Figure 5C. OCMC-MET alone increased AMPK and lowered mTOR, and it was even able to reduce the BCL2 level; however, under combined therapy, it significantly decreased both mTOR and BCL2 together. The GAPDH antibody is used as a loading control to confirm that protein loading is uniform across the gel. According to the literature [54], AMPK controls oxidative metabolism in cancer cells and downregulates mTOR to induce apoptosis. The prosurvival BCL2 family member was chosen for the study because it is regulated by glucose via the mTORC1 pathway. The fact that WZB117 monotherapy effectively reduced BCL2 levels suggests that the glycolysis pathway in cancer cells has been altered. The Western blot analysis clearly reveals the potential of WZB117-OCMC-MET to decrease BCL2, which is directly related to cancer cell glycolysis. WZB117-OCMC-MET inhibited BCL2 expression more effectively than OCMC-MET. The Western blot study proved that monotherapy of MET was ineffective against cancer since it increased AMPK but did not decrease BCL2; however, in combination, it targets both BCL2 and mTOR for more effective anticancer efficacy.

### 3.9. Cellular Uptake Assay by Confocal Microscopy to Evaluate the Efficacy of Conjugate Nanoparticles

To understand the cellular internalization efficacy of WZB117-OCMC-MET over OCMC-MET, both formulations were observed using CLSM after 2 h of incubation. MDA-MB-231 cells almost completely took up WZB117-OCMC-MET nanoparticles (Figure 5D) compared to OCMC-MET. We assume that higher WZB117-OCMC-MET uptake in MDA-MB-231 cells was induced by receptor-mediated endocytosis. Because WZB117 was able to reduce glucose concentrations in the cancer cell site, WZB117-OCMC-MET polymeric Nps facilitated cellular internalization better than OCMC-MET.

### 3.10. Cell Cycle Assay to Evaluate the Efficacy of Conjugate Nanoparticles

In general, cell replication necessitates the doubling of DNA and other biological components. The cell cycle is divided into four distinct phases defined by cell entry checkpoints: G1, S, G2, and M. The S phase is DNA synthesis. During G2, the cell prepares to divide, and division happens during M. External stimuli such as drugs, radiation, and reactive oxygen species induce DNA damage-related cell death. Figure 5E shows WZB117-OCMC-MET arresting cells in the G0-G1 phase. The increase in cells in the G0-G1 phase indicated the apoptosis induced by DNA damage. After 48 h, the WZB117-OCMC-MET treatment had a greater sub-G1 population than OCMC-MET (*p* < 0.05). The data clearly revealed that when combination therapy was delivered, WZB117 lowered glucose concentrations and stopped glycolysis, allowing the MET to inhibit mTOR and cause apoptosis better than the mono OCMC-MET therapy.

## 4. Conclusions

The study proved that OCMC polymeric Nps of MET decorated with WZB117 improved treatment efficacy by dual targeting the GLUT1 and mTOR pathways to alter cancer metabolism and improve BC management. A cytotoxicity study demonstrated that the monotherapy of MET in the OCMC polymeric formulation required a higher dose to induce cellular toxicity, which may have a negative impact on cancer management due to the hypoglycemic condition caused by the use of a high dose of MET. The Compusyn^®^ data also show that the WZB117-OCMC-MET formulation has a lower dose than the OCMC-MET formulation. The colony formation study indicated a long-term release profile for the WZB117-OCMC-MET, which also correlated with the in vitro release profile. DNA fragmentation, cellular internalization, and Western blot analysis demonstrate that the conjugated formulation outperforms monotherapy in terms of therapeutic efficacy. This polymeric Nps appears to be a cost-effective solution by using the repositioned safe-profile medication MET and the economical and easily available polymer OCMC. The formulation can be used to deliver the drug to the site of action, regardless of gender. WZB117-OCMC-MET targets cancer cell metabolism, so the formulation can also be used for other types of cancers that overexpress GLUT1 receptors. In future studies, the formulation might be used to test the effectiveness of an intraductal drug delivery system in an appropriate animal model. For underdeveloped and low-income nations, the technology would provide a superior approach to BC management in a safe and economical manner.

## 5. Patents

The synthesis process of the conjugation of WZB117 with OCMC described in the articles is patented (Patent No: 408278) under the Indian patent Act, 1970.

## Figures and Tables

**Table 1:** IC_50_ value and selectivity index of MET, OCMC-MET, WZB117-OCMC-MET, and WZB117 on MCF7, MDA-MB-231, and MCF10A cell lines.

Cell Line	IC_50_ (µg/mL) Value at 48 h			
	MET	OCMC-MET	WZB117-OCMC-MET	WZB117
MCF 7	>48.8	>46.2	10.2	17.7
MDA-MB-231	>55.5	>50.5	8.3	11.5
MCF 10A	>96.7	>98.6	93.3	78.1
SI for MCF10A/MCF7	1.98	2.4	9.1	4.5
SI for MCF10A/MDA-MB-231	1.75	2.1	11.2	6.7

## Data Availability

All data included in this study are available upon request by contact with the corresponding author.

## Supplementary Materials

The following supporting information can be downloaded at: https://www.mdpi.com/article/10.3390/polym15040976/s1, Figure S1: Pareto chart for Influence of independent variables on Particle size, Entrapment efficiency and Zeta potential, Box-Behnken Design of the 3D surface plot of independent variables on Particle size, Entrapment efficiency and Zeta potential; Figure S2: Particle size and zeta potential of OCMC-MET Nps. Table S1: % Swelling of OCMC-MET at different pH and time interval.
