# Supplementary material for: WZB117 Decorated Metformin-Carboxymethyl Chitosan Nanoparticles for Targeting Breast Cancer Metabolism
Source: Polymers (Basel). 2023 Feb 16;15(4):976. doi: 10.3390/polym15040976 (PMC9962472; doi:10.3390/polym15040976)
Supplement: Supplementary file 1 [file polymers-15-00976-s001.zip › polymers-2173405-supplementary.pdf]

Supplementary

WZB117 Decorated Metformin-Carboxymethyl Chitosan Nanoparticles for Targeting Breast Cancer Metabolism

Anindita De[1,*], Ashish Wadhwani[2], Sauraj[1], Parikshit Roychowdhury [3], Ji Hee Kang [1], Young Tag Ko [1] and Gowthamarajan Kuppusamy[3,*]

[1] College of Pharmacy, Gachon Institute of Pharmaceutical Science, Gachon University, Incheon 21936, Republic of Korea
[2] Faculty of Health Sciences, School of Pharmacy, JSS Academy of Higher Education and Research, Mauritius, Droopnath Ramphul St, Vacoas-Phoenix 73304, Mauritius
[3] Department of Pharmaceutics, JSS College of Pharmacy, JSS Academy of Higher Education & Research, Ooty 643001, Tamil Nadu, India
* Correspondence: aninditanirupa@gmail.com (A.D.); gowthamsang@jssuni.edu.in (G.K.); Tel.: +82-1098218750 (A.D.); +91-9443089812 (G.K.)

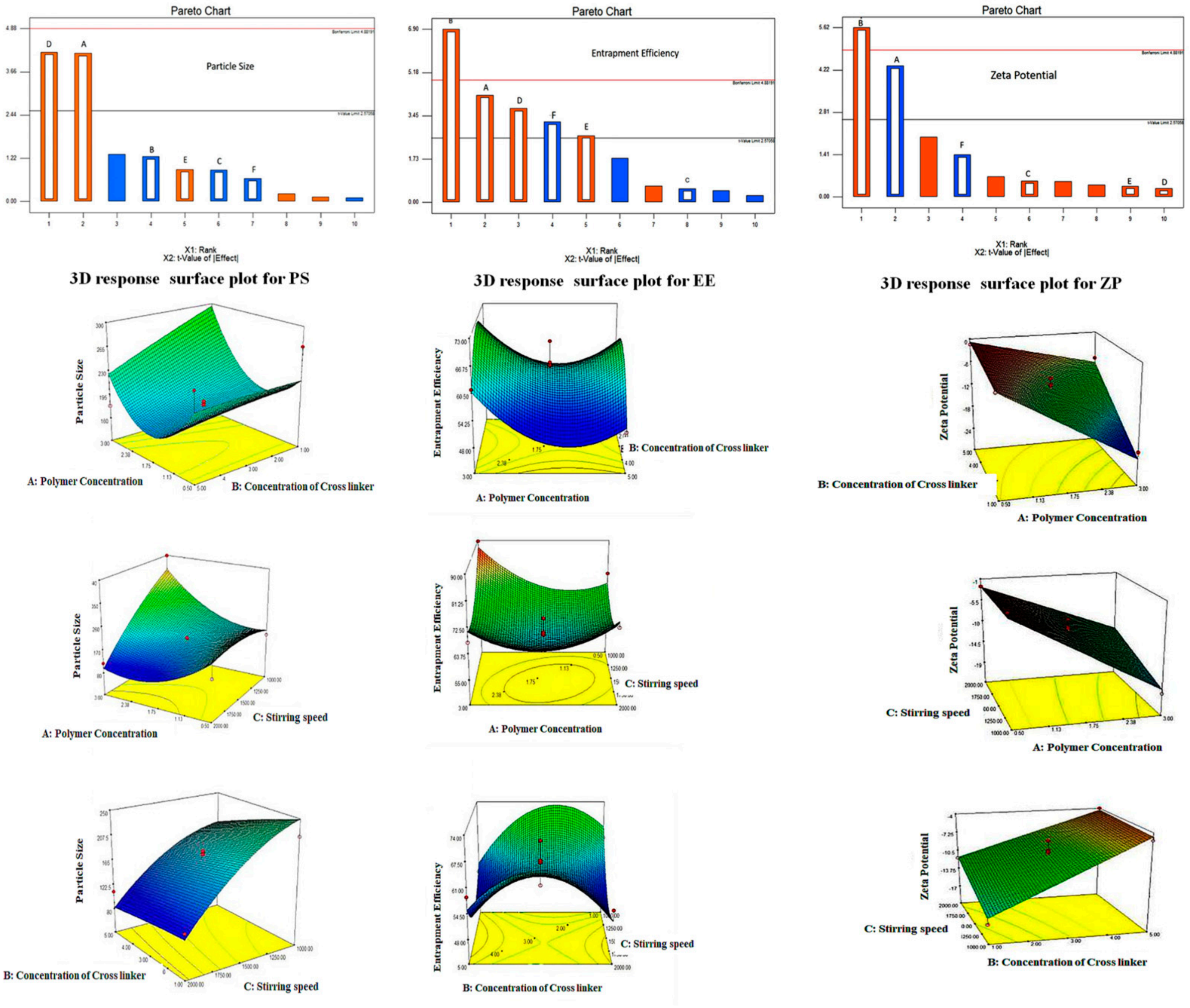

Figure S1. Pareto chart for Influence of independent variables on Particle size, Entrapment efficiency and Zeta potential, Box-Behnken Design of the 3D surface plot of independent variables on Particle size, Entrapment efficiency and Zeta potential.

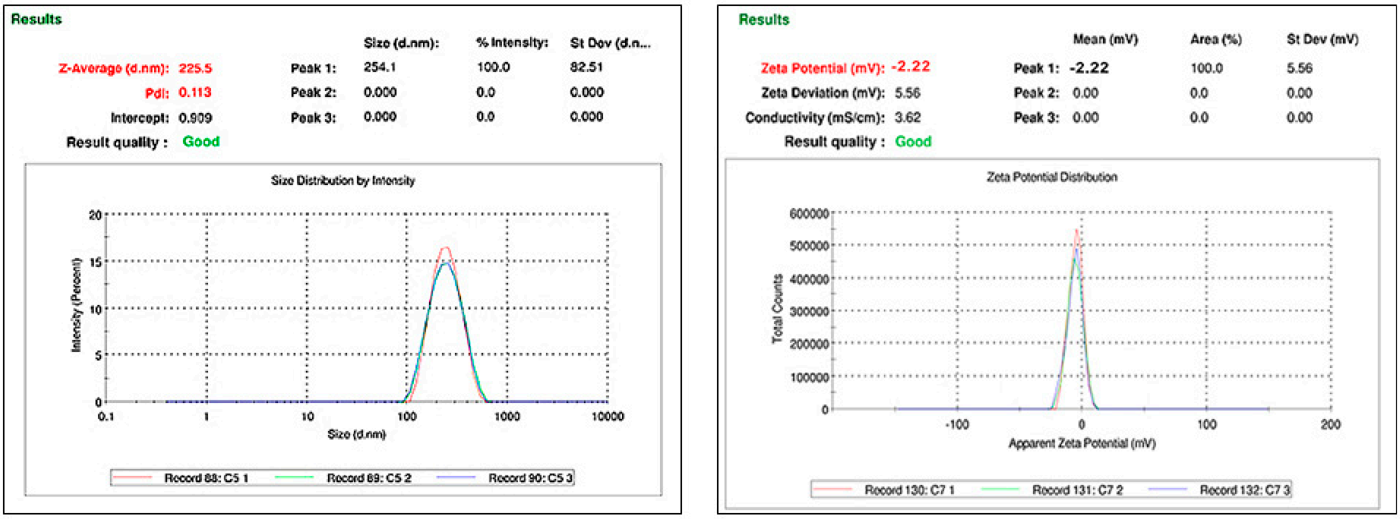

Figure S2. Particle size and zeta potential of OCMC-MET Nps.

Table S1: % Swelling of OCMC-MET at different pH and time interval.

Time (h)	% swelling at pH 5.5	% swelling at pH 7.4
0.5	10.33±1.1	1.50±0.42
1.0	43.23±6.56	10.0±2.56
5.0	76.66±16.8	32.23±5.6
